# Quantification of extracellular matrix remodeling for the non-invasive identification of graft fibrosis after liver transplantation

**DOI:** 10.1038/s41598-023-33100-7

**Published:** 2023-04-13

**Authors:** Bastian Engel, Ida Falk Villesen, Mette Juul Fisker Nielsen, Morten Karsdal, Richard Taubert, Elmar Jaeckel, Diana Julie Leeming

**Affiliations:** 1 Department of Gastroenterology, Hepatology, Infectious Diseases and Endocrinology, Hannover Medical School, Carl-Neuberg-Straße 1, 30625 Hannover, Germany; 2 Biomarkers and Research, Nordic Bioscience, Herlev, Denmark; 3 Present Address: Ajmera Transplant Centre, Toronto General Hospital, United Health Network, University of Toronto, Toronto, Canada

**Keywords:** Diagnostic markers, Predictive markers

## Abstract

Detecting patients with early post-transplant fibrosis after liver transplantation (LT) is very important. Non-invasive tests are needed to avoid liver biopsies. We aimed to detect fibrosis in liver transplant recipients (LTR) using extracellular matrix (ECM) remodeling biomarkers. ECM biomarkers for type III (PRO-C3), IV (PRO-C4), VI (PRO-C6) and XVIII (PRO-C18L) collagen formation and type IV collagen degradation (C4M) were measured by ELISA in prospectively collected, cryopreserved plasma samples (n = 100) of LTR with paired liver biopsies from a protocol biopsy program. Fibrosis ≥ F2 was present in 29% of patients (median 44 months post-LT). APRI and FIB-4 neither identified significant fibrosis nor were correlated with histopathological fibrosis scores, while ECM biomarkers (AUCs 0.67–0.74) did. The median levels of PRO-C3 (15.7 vs. 11.6 ng/ml; *p* = 0.002) and C4M (22.9 vs. 11.6 ng/ml; *p* = 0.006) levels were elevated in T-cell-mediated rejection compared to normal graft function. The median levels of PRO-C4 (178.9 vs. 151.8 ng/ml; *p* = 0.009) and C4M (18.9 vs. 16.8 ng/ml; *p* = 0.004) levels were increased if donor-specific antibodies were present. PRO-C6 had the highest sensitivity (100%), NPV (100%) and negative likelihood-ratio (0) for graft fibrosis. To conclude, ECM biomarkers are helpful in identifying patients at risk of relevant graft fibrosis.

## Introduction

End-stage liver disease and hepatocellular carcinoma (HCC) remain a global burden, with liver transplantation (LT) often being the last therapeutic option^1^. However, graft failure is the second leading cause of death in liver transplant recipients (LTR)^2^ often associated with post-transplant fibrosis, which was detected in protocol biopsies in more than 20% of patients not infected with hepatitis C virus (HCV) after LT^3,4^. Given the scarcity of donor organs it is critical to preserve graft function and prevent post-transplant cirrhosis in LTR. Early detection of not only fibrosis but also subclinical rejection or both recurrence and new onset of liver disease remains essential to ensure timely therapeutic intervention and to prevent the need for re-transplantation of LTR.

Although liver biopsy (LBx) is a safe procedure^5^, there is still a need for non-invasive methods to screen for the presence of relevant fibrosis in LTR. This could avoid the need for biopsy and allows more frequent serial measurements. Thus, fibrosis scores derived from readily available non-invasive tests (NITs) such as clinical parameters [aspartate aminotransferase/platelet ratio index (APRI), Fibrosis-4 score (FIB-4)], liver stiffness measurement (LSM), and biomarkers of extracellular matrix (ECM) turnover (e.g., Enhanced Liver Fibrosis (ELF)-test, the neoepitope markers PRO-C3, PRO-C4, etc.) are available.

NITs to detect significant fibrosis are widely used and validated in the pre-transplant period, as recommended by current guidelines^6^, and are accordingly transferred to patients after LT. LSM is commonly used for the non-invasive assessment of fibrosis. Both APRI and FIB-4 have been validated to detect fibrosis ≥ F2 in patients after LT, with LSM performing better than APRI and FIB-4, but data for the latter are relatively sparse and heterogeneous^7^.

The development of liver fibrosis involves remodeling and accumulation of ECM as a consequence of inflammation and activation of hepatic stellate cells. A major component of the ECM is collagens, and remodeling induces the release of fragments specific for either collagen formation or degradation. Protease cleavage of pro-peptides of immature collagens or disease-relevant protease degradation of the ECM releases fragments into the bloodstream, known as neo-epitopes, which can be measured by ELISAs^8,9^. The ability of these neo-epitope-specific collagen fragments to detect significant fibrosis has been studied in a variety of liver diseases prior to transplantation^10–12^.

To date, only one study has investigated the diagnostic ability of ECM biomarkers in LTR, including patients with HCV infection^13^. While they reliably identified patients with cirrhosis in the first year after LT, these biomarkers, measured in the first year after LT, could not identify patients with intermediate progression to post-transplant cirrhosis until the third to fifth year after LT. This group of patients was only identified when ECM biomarkers were measured closer to the development of fibrosis in the second or third year in this small cohort.

The aim of the current study was to investigate the diagnostic ability of ECM biomarkers for significant fibrosis and their correlation with histopathological assessment in a single-center cohort of patients after LT.

## Results

### Description of the study cohort

Of 100 LBx taken from 80 patients, 29 had significant fibrosis according to the Ishak fibrosis stage of ≥ F2, while 71 LBx did not have significant fibrosis. APRI and FIB-4 scores did not differ between the two groups. Alkaline phosphatase (ALP) and gamma glutamyl transferase (gGT) were significantly higher in the group with significant fibrosis than in the group of patients without significant fibrosis. Donor-specific antibodies (DSA) were present more frequently in patients in the fibrosis group. In the group without fibrosis, the biopsies more often showed no histological signs of rejection (NHR, representing a normal functioning liver graft) compared to the fibrosis group. No differences were observed in the presence of T cell mediated rejection (TCMR), subclinical T cell mediated rejection (subTCMR) and other graft injury (IND). RAI and mHAI scores were significantly higher in the fibrosis group. Of note, Tacrolimus (TAC) was used more frequently in the non-fibrosis group, while cyclosporine A (CsA) was used more frequently as the primary immunosuppressant in patients with fibrosis. The time between LBx and LT was longer in patients in the fibrosis group than in the non-fibrosis group. No differences were observed with regard to the reason for LT. Demographic data are summarized in Table 1.

**Table 1:** Patients’ demographics for the overall cohort, stratified according to presence of fibrosis ≥ F2 (Ishak fibrosis staging).

	All patients	Group F < 2	Group F ≥ 2	*p* value
Patient number	100	71	29	
Age (years) [median (range)]	51 (18–76)	50 (20–76)	57 (18–67)	0.05
Female sex (%)	37	35.2	41.4	0.65
Time after liver transplantation (months) [median (range)]	23 (2–298)	12 (2–152)	44 (5–298)	**0.001**
AST (times upper limit of normal) [median (range)]	0.8 (0.3–7.2) (n = 99)	0.8 (0.3–7.2) (n = 70)	1.0 (0.5–3.9)	0.21
ALT times upper limit of normal) [median (range)]	0.6 (0.1–11.8)	0.6 (0.1–11.8)	0.5 (0.2–5.2)	0.84
AP (times upper limit of normal) [median (range)]	0.9 (0.2–8.0) (n = 95)	0.8 (0.2–8.0) (n = 67)	1.2 (0.6–5.0) (n = 28)	**< 0.001**
gGT (times upper limit of normal) [median (range)]	1.3 (0.2–38.7)	0.8 (0.2–23.1)	1.8 (0.3–38.7)	**0.02**
Bilirubin (times upper limit of normal) ([median (range)]	0.6 (0.2–4.3)	0.5 (0.2–2.7)	0.6 (0.2–4.3)	0.29
Platelets (/nl) [median (range)]	177 (43–656)	177 (70–394)	178 (43–656)	0.92
Creatinine [µmol/l] (median (range))	96 (57–792) (n = 98)	99 (62–792) (n = 69)	93 (57–242)	0.39
APRI score (median (range))	0.5 (0.2–5.6) (n = 99)	0.5 (0.2–5.6) (n = 70)	0.5 (0.2–3.9)	0.46
FIB-4 Score (median (range))	1.4 (0.3–6.5) (n = 99)	1.4 (0.3–3.5) (n = 70)	1.6 (0.6–6.5)	0.14
Histopathological characteristics				
TCMR [n (%)]	14 (14)	11 (15.5)	3 (10.3)	0.75
subTCMR	39 (39)	25 (35.2)	14 (48.3)	0.26
Indeterminate [n (%)]	28 (28)	16 (22.5)	12 (41.4)	0.09
NGF [n (%)]	19 (19)	19 (26.8)	0 (0)	**0.001**
RAI [median (range)]	3 (0–8)	3 (0–6)	4 (1–8)	**0.03**
mHAI [median (range)]	3 (0–9)	2 (0–9)	4 (2–7)	**< 0.001**
DSA positive [n (%)]	45 (45)	25 (35.2)	20 (69.0)	**0.004**
Reason for LT				
AILD [n (%)]	34 (34)	26 (36.6)	8 (27.6)	0.49
Alcoholic [n (%)]	20 (20)	12 (16.9)	8 (27.6)	0.27
HCC [n (%)]	2 (2)	2 (2.8)	0 (0.0)	1.00
NASH [n (%)]	1 (1)	1 (1.4)	0 (0.0)	1.00
Viral [n (%)]	12 (12)	9 (12.7)	3 (10.3)	1.00
Cryptogenic [n (%)]	14 (14)	9 (12.7)	5 (17.2)	0.54
Other [n (%)]	17 (17)	12 (16.9)	5 (17.2)	
TAC [n (%)]	43 (43)	37 (52.1)	6 (20.7)	**0.004**
CSA [n (%)]	53 (53)	32 (45.1)	21 (72.4)	**0.02**
EVR [n (%)]	2 (2)	2 (2.8)	0 (0)	1.00
SIR [n (%)]	2 (2)	1 (1.4)	1 (3.4)	0.50

Significant *p* values for comparison between fibrosis and non-fibrosis group are designated in bold. Mann–Whitney-U Test was used for comparison of continuous variables and Fisher’s exact test was used for the comparison of categorial variables between these two groups.

### Test performance of ECM biomarkers

**Figure 1:**
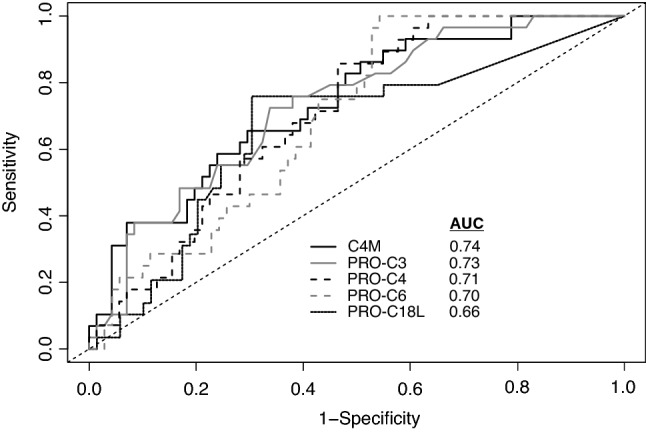
Diagnostic fidelity of different ECM biomarkers. Receiver operating curves of C4M (black line), PRO-C3 (grey line), PRO-C4 (dashed black line), PRO-C6 (dashed grey line) and PRO-C18L (dotted black line).

Several ECM biomarkers were measured in plasma samples from patients at the time of LBx to assess their diagnostic performance in detecting significant fibrosis. The AUC was used to assess the ability of the biomarkers to distinguish significant fibrosis of ≥ F2 from no fibrosis. C4M, PRO-C3, PRO-C4, PRO-C6, and PRO-C18L were able to distinguish significant fibrosis from non-fibrosis with moderate to good AUC (Fig. 1, Table 2). AUCs of C4M, PRO-C3, PRO-C4, PRO-C6 and PRO-C18L were not significantly different according to the DeLong test (Suppl. Table 1). The Youden index was used as a guide to determine the respective cut-off values to distinguish significant fibrosis and no fibrosis (Table 2).

**Table 2:** Summary of test performance of ECM biomarkers, APRI and FIB-4 for the detection of liver graft fibrosis.

	AUC	SE	95% CI	*p* value	Cut-Off (ng/ml)	Sensitivity (95% CI) (%)	Specificity (95% CI) (%)	Accuracy (95% CI) (%)	PPV (95% CI) (%)	NPV (95% CI) (%)	LR+ (95% CI)	LR− (95% CI)
C4M	0.74	0.05	0.63–0.84	**< 0.001**	19.0	65.5 (45.7–82.1)	**70.4 (58.4**–**80.7)**	**69.4 (59.4**–**78.2)**	36.5 (26.9–47.3)	**88.7 (82.3**–**93.0)**	2.22 (1.42–3.46)	0.49 (0.29–0.83)
PRO-C3	0.73	0.05	0.63–0.83	**< 0.001**	13.8	**72.4 (52.8**–**87.3)**	**66.2 (54.0**–**77.0)**	**67.5 (57.4**–**76.5)**	35.7 (27.2–45.2)	**90.2 (83.4**–**94.5)**	2.14 (0.23–0.77)	0.42 (0.23–0.77)
PRO-C4	0.71	0.05	0.61–0.81	**0.001**	155.3	**85.7 (67.3**–**96.0)**	53.5 (41.3–65.5)	60.2 (49.8–69.9)	32.4 (26.3–39.1)	**93.5 (85.0**–**97.4)**	1.84 (1.38–2.47)	0.27 (0.11–0.68)
PRO-C6	0.70	0.05	0.59–0.80	**0.003**	7.7	**100.0 (87.7**–**100.0)**	45.7 (33.7–58.1)	56.9 (46.5–66.9)	32.3 (27.8–37.2)	**100.0**	1.84 (1.49–2.28)	**0.00**
PRO-C18L	0.67	0.06	0.54–0.78	**0.01**	2.3	**75.9 (56.5**–**89.7)**	**69.6 (57.3**–**80.1)**	**70.9 (60.8**–**79.6)**	39.3 (30.0–49.4)	**91.7 (85.1**–**95.6)**	2.49 (1.65–3.76)	0.35 (0.18–0.67)
APRI	0.55	0.07	0.42–0.68	0.46	0.7	44.8 (26.5–64.3)	**75.7 (64.0**–**85.2)**	**69.4 (59.3**–**78.2)**	32.4 (21.2–46.1)	**84.1 (78.8**–**88.3)**	1.85 (1.04–3.29)	0.73 (0.51–1.04)
APRI cut-off 1					1.0	27.6 (12.7–47.2)	**81.4 (70.3**–**89.7)**	**70.3 (60.3**–**79.1)**	27.8 (15.2–45.4)	**81.3 (77.1**–**84.8)**	1.49 (0.69–3.2)	0.89 (0.69–1.14)
FIB-4	0.6	0.07	0.46–0.73	0.14	2.0	44.8 (26.5–64.3)	**78.6 (67.1**–**87.5)**	**71.6 (61.7**–**80.2)**	35.2 (22.9–49.8)	**84.6 (79.5**–**88.6)**	2.09 (1.14–3.83)	0.70 (0.49–1.00)
FIB-4 cut-off 1.45					1.45	51.7 (32.4–70.6)	52.9 (40.6–64.9)	52.6 (42.3–62.8)	22.2 (15.6–30.5)	**80.8 (73.2**–**86.7)**	1.10 (0.71–1.69)	0.91 (0.59–1.41)
FIB-4 cut-off 3.25					3.25	24.1 (10.3–43.5)	**95.7 (88.0**–**99.1)**	**81.0 (71.8**–**88.2)**	59.4 (28.9–84.0)	**82.9 (79.8**–**85.7)**	5.63 (1.56–20.29)	0.79 (0.64–0.98)

Sensitivity, specificity, accuracy, PPV, NPV, LR+ and LR− are shown including the 95% CI. If CIs for sensitivity, specificity, accuracy, PPV and NPV did not include 50%, values are depicted in bold. *p* values of AUC < 0.05 were regarded as significant and are depicted in bold.

*SE* standard error, *PPV* positive predictive value, *NPV* negative predictive value, *LR*+ positive likelihood-ratio, *LR−* negative likelihood-ratio.

### APRI and FIB-4 fail to identify significant fibrosis

**Figure 2:**
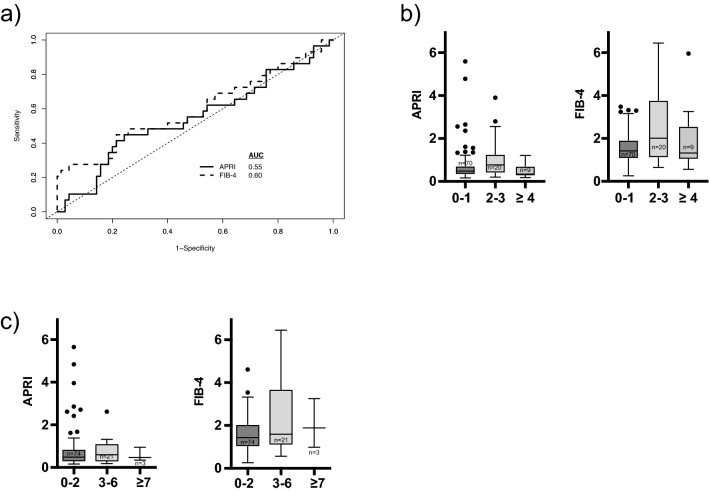
Diagnostic fidelity and correlation with histopathological scores of APRI and FIB-4. Receiver operating curves of APRI (solid black line) and FIB-4 (dashed black line) (**a**). Tukey Boxplots demonstrating APRI and FIB-4 in patients grouped according to their point values in Ishak fibrosis staging (**b**) and LAF-score (**c**). Kruskal–Wallis test and Bonferroni’s post-hoc test were used to compare groups.

In contrast, both the APRI and FIB-4 score could not distinguish significant fibrosis from no fibrosis based on the AUC (Fig. 2a and Table 2). Using published cut-off values of > 1 for APRI and > 3.25 for FIB-4 to identify individuals with a high likelihood of fibrosis resulted in a high to very high specificity of 81.4% and 95.7%, respectively, with a low sensitivity of 27.6% and 24.1% in our cohort. Specificity was significantly better for FIB-4 compared to APRI at these cut-offs. Using the published cut-off value of < 1.45 of the FIB-4 score to exclude significant fibrosis resulted in poor test performance. In addition, APRI and FIB-4 were not associated with Ishak fibrosis stage and liver allograft fibrosis (LAF) score (Fig. 2b,c). Test characteristics are summarized in Table 2.

### Comparison of ECM biomarkers to APRI and FIB-4

C4M and PRO-C3 were superior to APRI according to DeLong’s test (*p* = 0.03). None of the markers were significantly better than FIB-4. Similarly, PRO-C4, PRO-C6 and PRO-C18L were not significantly better than APRI (Suppl. Table 1).

Using the cut-offs identified, the highest sensitivity of 100% was found for PRO-C6 with a low specificity of 45.7%, a high NPV of 100% and an excellent negative likelihood-ratio of 0. The Sensitivity of PRO-C4 and PRO-C6 was superior to the multiple cut-offs of APRI and FIB-4. The highest specificity was found for C4M (70.4%) which had a moderate sensitivity of 65.5%. C4M and PRO-C18L were as specific as APRI and FIB-4, except when the cut-off of 3.25 for FIB-4 was used. The best AUCs with moderate to good sensitivity and specificity were found for C4M and PRO-C3. The results of the McNemar test comparing sensitivities and specificities of ECM biomarkers with those of the established fibrosis scores FIB-4 and APRI are shown in Supplementary Table 2. Using stepwise backward logistic regression, C4M and PRO-C3 were independently associated with the presence of significant fibrosis [odds ratios (95% confidence intervals) 1.135 (1.044–1.234) and 1.082 (1.014–1.153) respectively; corresponding *p* values 0.003 and 0.017]. A combination of both markers, where one point was awarded if the measured value was above the derived cut-off, did not provide any additional diagnostic value compared to any marker alone, except that the AUC was better than the one of FIB-4 (AUC 0.75, 95% confidence interval 0.64–0.85; *p* < 0.001) (Results of DeLong’s test are provided in Suppl. Table 1).

Overall, the ECM biomarkers were characterized by moderate to very high sensitivities (PRO-C3/4/6/18L) and low specificities (C4M, PRO-C3/6/18L), resulting in good to very good NPVs and clinically inappropriate low PPVs (Table 2).

### Association of biomarkers with histological parameters

**Figure 3:**
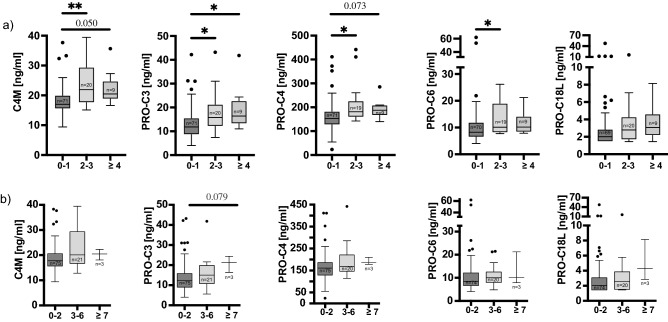
Correlation of ECM biomarkers with histopathological scores of fibrosis. Tukey Boxplots demonstrating differences in median biomarker levels in patients grouped according to points in Ishak fibrosis staging (**a**) and LAF-score (**b**). Kruskal–Wallis-test with Bonferroni’s post-hoc test were used for comparison between groups. *indicates *p* < 0.05; **indicates *p* < 0.01.

In contrast to APRI and FIB-4, all biomarkers except PRO-C18L were elevated in mild fibrosis (Ishak stages F2 and F3) compared to those without significant fibrosis (Fig. 3a). For fibrosis ≥ F4, biomarker levels were further increased for PRO-C3, while slightly missing the significance level for C4M and PRO-C4. However, the group size for patients with fibrosis ≥ F4 was small. There was a trend towards higher biomarker levels according to LAF score, although statistical tests were likely influenced by the small group size for a LAF score ≥ 7 (Fig. 3b).

### Association of biomarkers with clinical parameters

**Figure 4:**
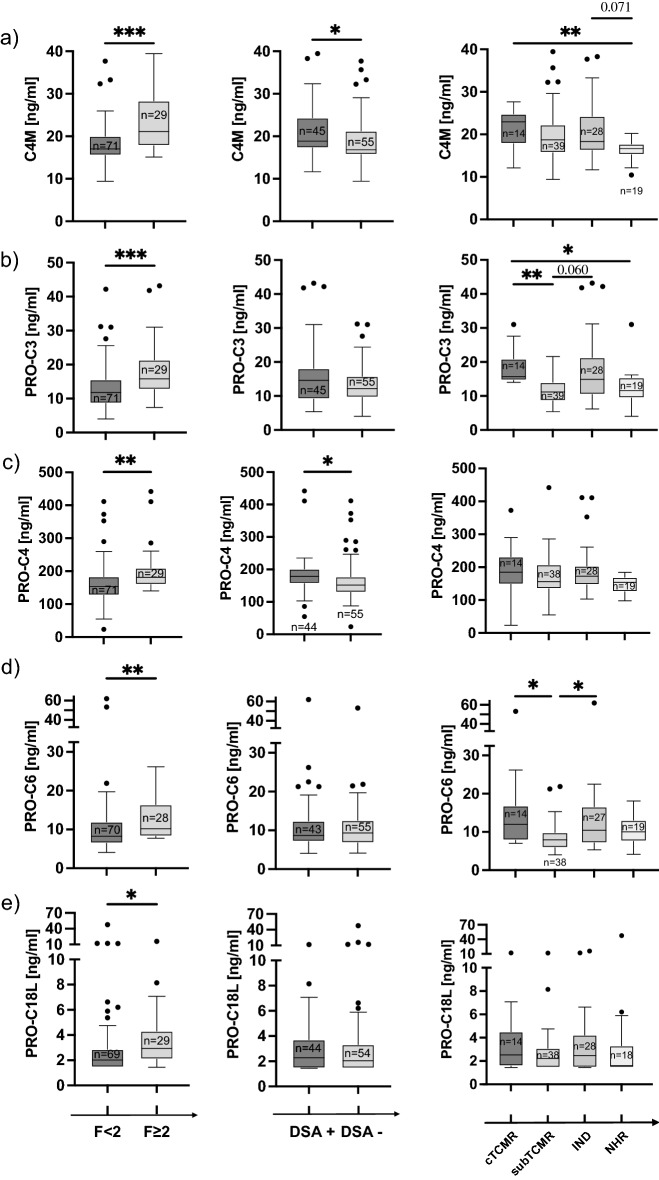
Correlation of ECM biomarkers with clinical phenotypes. Tukey Boxplots showing differences in biomarker levels between significant fibrosis (≥ F2) and no significant fibrosis (< F2) (left panel), differences between DSA positive patients (DSA+) and DSA negative (DSA−) patients (middle panel) and differences in biomarker levels based on histopathological classification of T cell-mediated rejection (TCMR), subclinical T cell-mediated rejection (subTCMR), other graft injury (IND) and no histological rejection (NHR). Differences are shown for C4M (**a**), PRO-C3 (**b**), PRO-C4 (**c**), PRO-C6 (**d**) and PRO-C18L (**e**). Two groups were compared using Mann–Whitney-U test. More than two groups were compared using Kruskal–Wallis test and Bonferroni’s post-hoc test. *indicates *p* < 0.05; **indicates *p* < 0.01; ***indicates *p* < 0.001.

Median C4M levels were higher in patients with fibrosis ≥ F2 than in patients without significant fibrosis (21.1 vs. 17.0 ng/ml; *p* < 0.001). Median C4M levels were increased in patients with TCMR compared with patients with NHR (22.9 vs. 16.6 ng/ml; *p* = 0.006) and were higher in patients with DSA (18.9 vs. 16.8 ng/ml; *p* = 0.004) (Fig. 4a).

Median PRO-C3 levels were increased in patients with significant fibrosis (15.8 vs. 11.8 ng/ml; *p* < 0.001) and with TCMR (15.7 ng/ml) compared with subTCMR (11.2 ng/ml; *p* = 0.002) and NHR (11.6 ng/ml; *p* = 0.01) but were not associated with the presence of DSA (Fig. 4b).

Median levels of PRO-C4 increased in patients with significant fibrosis (181.5 vs. 153.5 ng/ml; *p* = 0.001) and in the presence of DSA (178.9 vs. 151.8 ng/ml; *p* = 0.009), but were not associated with any type of rejection (Fig. 4c).

Median levels of PRO-C6 increased in patients with significant fibrosis (10.2 vs 8.2 ng/ml; *p* = 0.003) and patients with TCMR (12.0 ng/ml) and IND (10.4 ng/ml) compared to subTCMR (7.9 ng/ml; *p* = 0.03 and *p* = 0.04, respectively), but did not differ depending on the presence of DSA (Fig. 4d).

Median levels of PRO-C18L increased in patients with significant fibrosis (2.9 vs. 2.0 ng/ml; *p* = 0.01). They were not associated with any rejection subtype or the presence of DSA (Fig. 4e).

For all biomarkers, median levels in patients treated with TAC were not significantly different from those treated with CsA as the main immunosuppressive therapy (Suppl. Fig. 1). Biomarker levels did not differ significantly when patients were stratified by reason (Suppl. Fig. 2a–c,e) except for PRO-C6 levels (Suppl. Fig. 2d), which were higher in patients transplanted for alcoholic liver diseases than in patients transplanted for autoimmune liver diseases. However, PRO-C6 levels in alcoholic liver disease were not different from all other reasons for LT, which had levels similar to those of autoimmune liver diseases (Suppl. Fig. 2d). Both the Ishak fibrosis stage and the LAF score were similar in all groups (Suppl. Fig. 2f,g).

### Stable test performance in patients without relevant inflammation

As median levels of C4M, PRO-C3 and PRO-C6 were elevated in TCMR and non-invasive tests are preferably used in a stable setting after LT for screening purposes, we performed subgroup analysis on 77 samples from patients without relevant elevation of liver enzymes (alanine amino-transferase (ALT), aspartate amino-transferase (AST) and alkaline phosphatase (ALP) < 2 × upper limit of normal).

In total, there were 56 cases without significant fibrosis and 21 cases with fibrosis ≥ F2. As in the overall cohort, ALP, gGT, RAI and mHAI were higher in the fibrosis group. Median AST was higher in patients with significant fibrosis, but within the normal limits. DSA occured more frequently in the fibrosis group and sampling was later after LT in the fibrosis group. Patients in the group without fibrosis more often as their primary immunosuppressant. The median levels of C4M, PRO-C3, PRO-C4 and PRO-C6 were significantly different between patients with significant fibrosis and without fibrosis, even in this subcohort of patients with normal or near normal liver enzymes. The data are summarized in Supplementary Table 3. In this cohort, C4M, PRO-C3, PRO-C4 and PRO-C6 were able to significantly discriminate between significant fibrosis and non-fibrosis with moderate to good AUC, while PRO-C18L, APRI and FIB-4 did not. The performance characteristics of the assays were comparable to the overall cohort using the originally established cut-off values. In particular, PRO-C6 maintained its superior sensitivity to all other tests, while FIB-4 with a cut-off of 3.25 and APRI with a cut-off of 1 were most specific. PRO-C4 was more sensitive than APRI regardless of the cut-off used, while slightly missing significance for superior sensitivity to FIB-4 using a cut-off of 1.45. The data are summarized in Supplemental Table 4.

### Test performance is independent of time after transplantation

**Figure 5:**
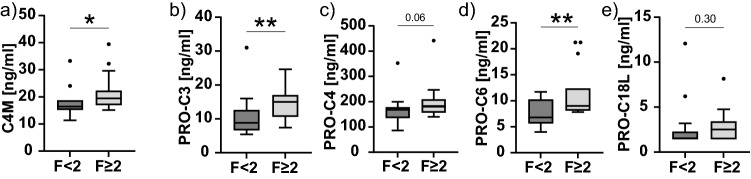
ECM biomarker levels in relation to fibrosis in a propensity-score matched cohort for graft age. Tukey Boxplots showing differences in biomarker levels between significant fibrosis (≥ F2) and no significant fibrosis (< F2). Each group consisted of 15 patients. Patients were matched using propensity-score matching for time after transplantation in months. Two groups were compared using Mann–Whitney-U test. *indicates *p* < 0.05; **indicates *p* < 0.01.

Patients with fibrosis presented significantly later after LT than patients without fibrosis (Suppl. Table 3). To exclude an influence of mere graft age on ECM biomarkers, we performed propensity score matching for post-transplant time in all patients with liver enzymes below 2× ULN (Supplementary Fig. 3). Fifteen LTR without significant fibrosis were matched with 15 LTR with fibrosis ≥ F2. The median time post-transplant was 25 months in both groups. LTR with fibrosis had higher histological inflammatory activity despite similar levels of transaminases. Demographic data are summarized in Suppl. Table 5. Nevertheless, C4M, PRO-C3 and PRO-C6 levels were significantly higher in patients with at least F2 Fibrosis than in those without fibrosis (Fig. 5A–C). For PRO-C3, significance was marginally missed (Fig. 5D). PRO-C18L levels did not differ between patients with and without fibrosis in this group of LTR with comparable time after LT (Fig. 5E).

### Long-term follow-up of fibrosis development

Follow-up data on fibrosis development between the third and fifth year after LT were available for 26 patients who had samples taken within the first year after LT. Eight patients developed fibrosis ≥ F2 during this period, while 18 patients did not develop significant fibrosis until 5 years after LT. Patients who developed fibrosis had significantly higher gGT levels at baseline and were more likely to have histological findings indicating IND. Demographic data are summarized in Supplementary Table 6. Long-term follow-up of patients with fibrosis in these 26 patients showed decreased fibrosis from baseline to year three to five in six patients, stable fibrosis in 16 patients, and increased fibrosis in four patients. However, neither ECM biomarkers nor APRI and FIB-4 differed between these groups and were therefore not prognostic in this small study (Supplementary Fig. 4). Similarly, serial sampling was available in 15 patients. Two patients had more than one follow-up sample available, but their fibrosis stage did not change, so only the first follow-up sample was considered in all patients. Only four patients progressed to fibrosis ≥ F2, one patient experienced regression of fibrosis and 10 patients had stable fibrosis stage. None of the ECM biomarkers reflected progression in this very small subgroup (Suppl. Fig. 5).

## Discussion

In the past, rapidly progressive fibrosis and post-transplant cirrhosis were observed in HCV-infected patients after LT^14^. Since post-LT patients are now usually HCV RNA negative, there are still a variety of reasons for significant fibrosis to occur, such as recurrent and de novo liver disease or smouldering alloimmune response driving fibrogenesis. Data from surveillance biopsy programs indicate a prevalence of relevant fibrosis (≥ F2) in 20–25% of patients, not only within the first year, but especially in long-term survivors^4,15^. LBx is still considered the gold standard for detecting fibrosis in LTR and also serves other purposes, e.g., quantification of inflammation, detection of rejection or disease recurrence, etc., but the performance of surveillance biopsies has been criticized because of periprocedural risk and cost, but also because of sampling bias and the fact that LBx itself is an imperfect gold standard^16,17^. At least, the procedural risk of LBx is fortunately low, though not negligible^4,5^. Although non-invasive tests to assess significant liver fibrosis in the pre-transplant period have been extensively validated and recommended by an EASL guideline^6^, non-invasive assessment of fibrosis after LT is less well studied and still in its infancy in terms of day-to-day clinical application, although there is a need for non-invasive tests for the management of LTR. Of all the non-invasive tests, LSMs such as transient elastography are studied best after LT and perform better than APRI and FIB-4 to detect liver graft fibrosis^7^. It should be noted that most of the data are from the HCV era and more recent evidence on non-invasive tests is relatively scarce. It is critical that a non-invasive test can confidently identify patients without evidence of fibrosis to omit or defer LBx in these patients. Although LSM has been shown to safely identify patients with post-transplant cirrhosis, the accuracy was lower to identify patients with earlier stages of fibrosis^6^. While the measurement of liver stiffness, and also histology, are rather static tools that help to assess the consequences of graft injury, ECM biomarkers, as discussed for other markers^18^, provide a more dynamic assessment of the fibrotic response to injury or inflammation and correlate closely with the severity of liver disease by measuring both the formation and degradation of ECM protein fragments in plasma^19^. In this regard, ECM biomarkers as a dynamic tool to assess ongoing fibrogenesis induced by graft injury or inflammation, could complement the diagnostic work-up of LTR and help to identify patients with moderate fibrosis and ongoing necroinflammatory activity sustaining fibrogenesis who could benefit from modification of immunosuppressive management. At our center, immunosuppression is tailored to individual patient needs after assessing graft injury by surveillance liver biopsies as recently published^4^. After thorough exclusion of non-alloimmunological causes of graft injury, immunosuppression is tapered in individuals at low risk of alloimmune injury (i.e. no inflammation and fibrosis in the histological assessment), is left as it is in cases with some inflammation but without fibrosis and is increased with MMF being substituted by everolimus in patients with high risk, namely inflammation and fibrosis in the histological assessment and the presence of DSA. Everolimus has shown an anti-fibrotic effect in experimental studies and thus may be of benefit to patients identified by ECM biomarkers, especially those with still moderate, potentially reversible fibrosis (Fig. 3)^20–22^.

Our study explores the potential capability of ECM biomarkers as promising surrogates for non-invasively assessing the presence of relevant graft fibrosis and ongoing fibrogenesis. To date, only one study on ECM biomarkers has been published in patients after LT^13^. In this relatively small cohort, which included patients with HCV infection, C4M, PRO-C3, and PRO-C4 were measured in samples up to 3 years after LT. ECM biomarkers measured in the first year identified patients who developed cirrhosis within the first year, while they did not identify patients who developed cirrhosis in the third to fifth year. However, in blood samples from 2 and 3 years after LT, C4M, PRO-C3, PRO-C4 and PRO-C5 were also elevated in patients who developed significant fibrosis between the third and fifth year compared to those who did not develop fibrosis. It should be noted that this study examined patients with rapid development of liver graft cirrhosis, which is a much rarer kinetic of fibrosis development after LT than the appearance of fibrosis after the first year.

In that sense, our study could not validate the published results because the rate of rapidly occurring post-transplant cirrhosis was low, as we did not include patients with HCV-infection, which has been a cause of early post-transplant cirrhosis. However, we identified a subgroup of patients who had plasma samples and paired LBx within the first year and progressed to significant fibrosis in the third to fifth year. As published, we were also unable to predict intermediate progression to significant fibrosis based on these ECM biomarkers measured within the first year after LT, and the number of patients with serial sampling was too small for predictive analysis. For the second and third year after LT, we had five samples from patients with significant fibrosis and 15 samples from patients without fibrosis. However, follow-up of these patients was not stringently available for the third to fifth year, which prevents validation of published results for this subgroup in our cohort.

The strength of our current study lies in the use of protocol biopsies as the gold standard to assess fibrosis and inflammation. Including patients from a protocol biopsy program captures patients with early fibrotic changes that might be missed by transient elastography^7^. Of note, ECM biomarkers have been shown primarily to identify patients with early fibrotic changes^23^ who could potentially benefit from targeted therapeutic interventions, particularly an increase or change in immunosuppression as described above.

We demonstrated that specific ECM biomarkers, compared to APRI or FIB-4, were able to identify or exclude significant fibrosis at any time point after LT, from early (2–5 months) to very late (up to 298 months). The timing of sampling covers a wide interval in patients with fibrosis, and different causes with different kinetics may underlie at different time points after LT. Nevertheless, ECM biomarkers correlate robustly with histological scoring of fibrosis in this heterogeneous group, are not relevantly influenced by the original reason for LT, and help to identify patients with relevant graft fibrosis regardless of graft age in a sub-cohort of patients matched for this covariate. It is noticeable, that TAC was used less frequently in patients with fibrosis ≥ F2 than in patients without fibrosis. The switch from CsA to TAC as the primary CNI was made several years ago at our center because of evidence of reduced mortality, graft loss and acute rejection episodes in patients on TAC as the primary immunosuppressant^24,25^. Nevertheless, CsA is continued in patients with stable graft function without evidence of graft injury. As for differences in the development or progression of fibrosis in patients on TAC compared to CsA, there is limited evidence. In a rat model, no differences in fibrosis development were found between TAC and CsA^22^, and severe fibrosis occurred in hepatitis C virus positive patients on TAC or CsA with similar frequencies 1 year after LT^26^. However, patients in the fibrosis group had more severe subclinical inflammation, which may also be related to a lower efficacy of CsA compared to TAC, further fueling the ongoing debate about the importance of subclinical graft injury for long-term graft outcome. As the use of TAC reduces the risk of acute rejection episodes compared to CsA^24^, it may well be that it also reduces subclinical inflammation, but most transplant centers do not perform protocol LBx for graft monitoring, as our center does, which prevents a large-scale multicenter evaluation of this hypothesis. However, dynamic markers such as ECM biomarkers help identify patients with ongoing fibrogenesis and could therefore be helpful to identify patients with subclinical graft injury progressing to fibrosis, thus justifying their use in future multi-center studies.

Our study also shows that protocol biopsies remain a useful tool, as the classical fibrosis screening scores FIB-4 and APRI have low sensitivity for the detection of liver graft fibrosis and ECM biomarkers are still explored in the transplant setting but not regularly used in daily clinical practice. The inferior performance of APRI and FIB-4 was most likely because one of the main components of both scores, the platelets, are persistently reduced after transplantation, because of an incomplete normalization of spleen size after transplantation, and immunosuppressive antimetabolites are frequently given to LTR. However, APRI and FIB4 had a good specificity and negative predictive value even after LT. A limitation of our study is that LSM was not available for direct comparison in most patients. However, LSM was not required for clinical care as the patients received surveillance biopsies to monitor the graft.

The evaluation of diagnostic capabilities and the establishment of cut-off values for ECM biomarkers for different stages of fibrosis are limited in our study by a small sample size of the total cohort and the small number of patients with higher stages of fibrosis. However, as a proof-of-concept, we wanted to examine the potential utility of ECM biomarkers as diagnostic tools for fibrosis in LTR. We used the Youden Index that maximizes both sensitivity and specificity to avoid potential bias by artificially maximizing sensitivity, which may be more useful in a clinical scenario where these markers are used as screening tools. However, a combination of markers did not result in improved diagnostic capacities compared to one marker alone.

Even with this approach, PRO-C6 provided excellent sensitivity at the expense of specificity in our cohort. It correctly identified all patients with fibrosis ≥ F2 while misidentifying about half of patients without fibrosis, representing a potential clinical benefit as 50% of patients could defer biopsies if they were used to detect fibrosis. As seen in our cohort and others^27–30^, patients with significant fibrosis had more histological inflammation and presence of DSA despite comparable transaminases, suggesting a greater number of patients who could benefit from LBx, not only in terms of detecting fibrosis but also in assessing inflammation, which cannot be replaced by measuring ECM biomarkers. These findings, inflammation and presence of DSA, may also be the main reason for the lower specificity of ECM biomarkers for detecting fibrosis. However, these markers performed equally well both in the general cohort and in the cohort of patients with only subclinical inflammation, so they may be suitable for screening purposes in different clinical settings pending validation in a larger cohort. The presence of DSA can also be used as a non-invasive marker of liver graft injury, including fibrosis, although it has traditionally been associated with ABMR^15^. In this context, DSA positivity has a high predictive accuracy for the presence of graft injury, while absence of DSA is not indicative of the presence or absence of graft injury due to the low overall frequency of DSA in many liver transplant cohorts. The ELF-test has recently been approved by the FDA for fibrosis screening as it works well in various liver diseases^31–33^. However, in a systemic review^33^, the test showed high sensitivity but low specificity for detecting significant fibrosis in non-alcoholic fatty liver disease in the range of PRO-C6 in our study. In addition, there are no data on the ELF test for LTR. Whether these markers will be widely available and a cost-effective tool, e.g. compared to expensive LSM devices or the ELF test, depends on future clinical validation studies.

In summary, we have explored the utility of ECM biomarkers as surrogates for ECM remodeling and fibrosis in LTR and provided a potential outlook on their respective diagnostic capabilities. ECM biomarkers offer a very high NPV with low specificity and correlate well with histological staging of fibrosis. Normal levels can safely exclude liver graft fibrosis, but liver biopsies may still be required in patients with elevated levels. Our study was a proof of concept and a definitive judgment of clinical utility is beyond the scope of our study, so the investigation of ECM biomarkers in a larger, more homogeneous cohort of LTRs and their longitudinal evaluation are still pending. The final clinical use case will be determined by such a subsequent study.

## Methods

### Patient cohort

Eighty patients with 100 available plasma samples (EDTA-plasma) paired to LBx were recruited from an ongoing prospective liver allograft biorepository from Hannover Medical School, Germany and analyzed retrospectively. All adult LTR that agreed to participate and had at least one LBx, were included in our prospective liver allograft biorepository from 2008 to 2019. Study participation in case of biopsy was also offered to patients transplanted prior to 2008. Liver transplantation in patients took place between 1990 and 2017. Patients with replicative viral hepatitis after LT or signs of recurrence of the underlying liver disease/cause of transplantation were not included. Patients were included both for surveillance LBx (to guide immunosuppressive treatment) and indication LBx (e.g., suspicion of rejection or significant graft fibrosis). Participation was voluntary and rejection of participation did not influence clinical management. All patients provided written informed consent. Only patients with available paired liver tissue and plasma sample at the same time point were included. Plasma samples were collected within a 24-h time frame around a LBx and were cryo-conserved at − 80 °C. The study was approved by the local Ethics Committee (protocol number 933 for project Z2 of comprehensive research center 738; MHH Ethikkommission; Hannover Medical School, Hannover, Germany). The study conforms to the ethical guidelines of the 1975 Declaration of Helsinki.

### Biochemical measurements

Transaminases, cholestasis parameters, total blood count and creatinine were extracted from patients’ charts and originally analyzed using routine clinical laboratory high-throughput methods. Donor specific antibodies were assessed from stored plasma samples, if they were not analyzed during collection of the LBx already. In short, samples were screened using mixed HLA antigen-charged polysterene beads (LIFECODES LifeScreen Deluxe-LMX test Gen-Probe-Immucor, Stanford, CT, USA) and a multichannel flow array (Luminex, Austin, Tx) as previously described^34^ and in accordance with the manufacturer’s instructions. Positive screening results led to differentiation by class I/class II single-antigen beads (LIFECODES Single Antigen-LSA test Gen-Probe Immucor, Stanford, CT, USA).

FIB-4 and APRI Score were calculated as published^35,36^.

### Histological assessment

Formalin-fixed, 2 µm thin sections of liver tissue were stained using hematoxylin and eosin, elastic van Gieson, periodic acid-Schiff, silver, Berlin blue and rhodamine stain. Examination and scoring were performed by experienced liver pathologists in a blinded manner. Rejection activity index (RAI)^37^, modified histological activity index (mHAI)^38^ and the LAF^39^ score were assessed as published. The mHAI according to Ishak et al. was used given its widespread distribution and application both pre- and post LT, while the LAF score was added as a score that was specifically designed to assess graft fibrosis after LT. At least moderate fibrosis was defined as periportal fibrosis (Ishak F)^38^ ≥ 2. SubTCMR and cTCMR were defined as recently published^40^. In short, TCMR for the diagnosis of subTCMR and cTCMR was defined by a Banff RAI ≥ 1 + 1 + 1 to exclude borderline TCMR. IND comprised a group of patients with histopathological graft injury not belonging to a distinct entity like cTCMR or subTCMR.

### Biomarkers of ECM remodeling

To assess ECM remodeling, blood samples were analyzed using the biomarkers PRO-C3, PRO-C4, C4M, PRO-C6, and PRO-C18L by ELISA at Nordic Bioscience. A competitive ELISA method with monoclonal antibodies detection was used. Ninety-six well streptavidin plates coated with biotinylated synthetic peptide were dissolved in an optimized assay and incubated for 30 min at 20 °C. A calibrator peptide amount of 20 µL or an appropriate dilution of analyte was added into the wells. This included 100 µL horseradish peroxidase conjugated with monoclonal antibodies directed against the specific sequence of interest and incubated for one or 20 h at 4 °C or 20 °C, depending on the assay. One hundred µL tetramethylbenzidine (Kem‐En‐Tec cat. 4380H) was added and incubated for 15 min at 20 °C in the dark. Sulfuric acid (100 µL, 1%) was used to stop reactions for measurements at 450 nm and with reference measurements performed at 650 nm. Samples were centrifuged at 300 rpm at incubation. Plates were washed five times after coating and sample incubation in a washing buffer (20 mmol/L Tris, 50 mmol/L NaCl, pH 7.2). A four-parametric fit model was used as calibration curves. For measurements below or above the lower- and upper limit of measurement range, values were recorded as the lowest or highest value within the detection range of the specific assay, respectively.

The biomarkers PRO-C3, PRO-C4, PRO-C6 and PRO-C18L measure formation of type III collagen, type IV collagen, type VI collagen and type XVIII collagen long isoform, respectively. C4M measures degradation of type IV collagen.

### Statistics

Statistical analyses were performed using IBM SPSS Statistics version 27, RStudio 2021.9.0.351 “Ghost Orchid” and GraphPad Prism 9.4.0. For non-parametric continuous variables, the Mann–Whitney-U test was used to compare quantitative data between two independent groups and the Kruskal–Wallis test with Bonferroni’s multiple comparison post-hoc test was used for comparison between more than two groups. The Fisher’s exact test was used to compare contingency tables with two groups. Binary logistic regression was performed with the stepwise-backwards method. The AUC was calculated to assess diagnostic performance of the biomarkers and the Youden’s Index was used to guide identification of respective cut-off values. AUCs were compared using DeLong’s test^41^ with easyROC^42^. For calculating accuracy, negative predictive value (NPV) and positive predictive value (PPV), the mean prevalence of periportal fibrosis ≥ F2 at our center was calculated from previously published reports^15,40^. Propensity score matching was performed with the MatchIt package using the nearest neighbor method, a 1:1 ratio and a caliper of 0.25^43^. *p* values below 0.05 were considered statistically significant.

## Supplementary Information

Supplementary Information.

## Data Availability

The data that support the plots within this paper and other findings of this study are available from the corresponding author upon reasonable request.

## Abbreviations

AILD: Autoimmune liver disease
ALP: Alkaline phosphatase
ALT: Alanine aminotransferase
APRI: Aspartate aminotransferase/platelet ratio index
AST: Aspartate aminotransferase
CsA: Cyclosporine A
DSA: Donor specific antibodies
ECM: Extracellular matrix
EVR: Everolimus
FIB-4 score: Fibrosis-4 score
gGT: Gamma-glutamyltransferase
HCC: Hepatocellular carcinoma
HCV: Hepatitis C virus
IND: Other graft injury
LAF: Liver allograft fibrosis
LBx: Liver biopsy
LSM: Liver stiffness measurement
LTR: Liver transplant recipients
LT: Liver transplantation
mHAI: Modified histological activity index
NHR: No histological rejection
NPV: Negative predictive value
PPV: Positive predictive value
RAI: Rejection activity index
SIR: Sirolimus
subTCMR: Subclinical T cell mediated rejection
TAC: Tacrolimus
TCMR: T cell mediated rejection
