# Supplementary material for: Quantification of extracellular matrix remodeling for the non-invasive identification of graft fibrosis after liver transplantation
Source: Sci Rep. 2023 Apr 13;13:6103. doi: 10.1038/s41598-023-33100-7 (PMC10101979; doi:10.1038/s41598-023-33100-7)
Supplement: Supplementary file 1 — Supplementary Information. [file 41598_2023_33100_MOESM1_ESM.pdf]

Quantification of extracellular matrix remodeling for the non-invasive identification of graft fibrosis after liver transplantation

*Bastian Engel[1], Ida Falk Villesen[2], Mette Juul Nielsen[2], Morten Karsdal[2], #Richard Taubert[1], #Elmar Jaeckel[1, 3], #Diana Julie Leeming[2]

# authors share last authorship

* corresponding author

Supplemental figures

Supplemental Figure 1

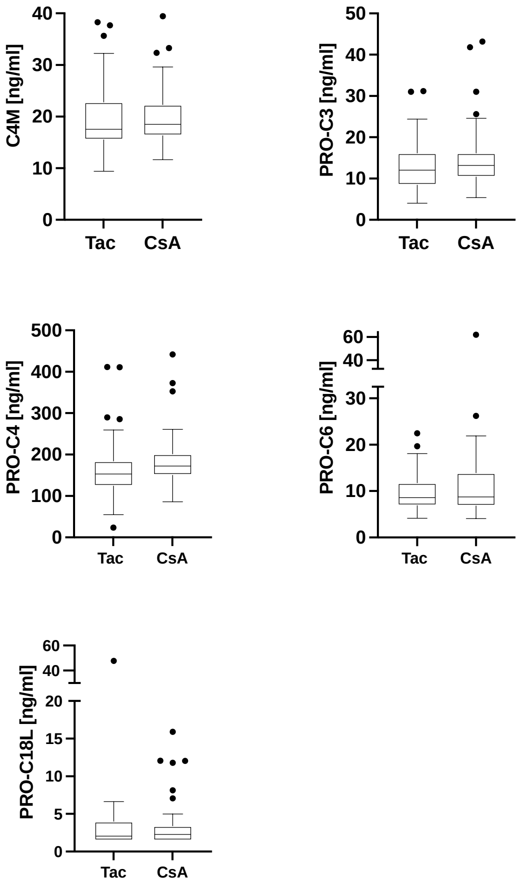

Supplemental figure 1: Effect of CNI-choice on ECM biomarker levels

Tukey Boxplots showing biomarker levels for patients with tacrolimus (Tac) or cyclosporine A (CsA) as primary immunosuppressant. The Mann-Whitney-U test was used for group comparison. Differences between groups were not significant. N=41 in Tac group (except for PRO-C6: n=40); n=47 in CsA group (exceptions: PRO-C4&PRO-C18L: n=46).

Supplemental Figure 2

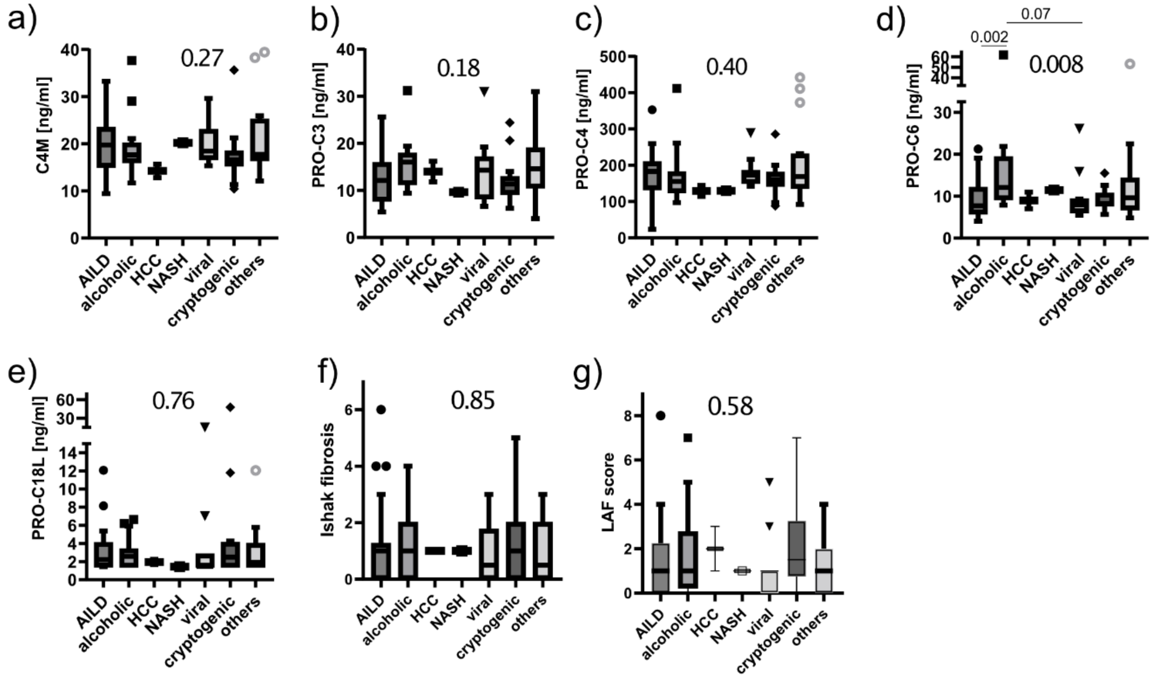

Supplemental figure 2: Effect of reason for liver transplantation on ECM biomarker levels and fibrosis stage

Tukey Boxplots showing biomarker levels (a-e), Ishak fibrosis stage (f) or LAF score (g) dependent on reason for LT. Numbers indicate the p-value of the Kruskal-Wallis Test. In panel d, Kruskal-Wallis Test was significant and the significant subgroup comparison (Bonferroni adjusted) is shown above the respective line.

Supplemental Figure 3

a)

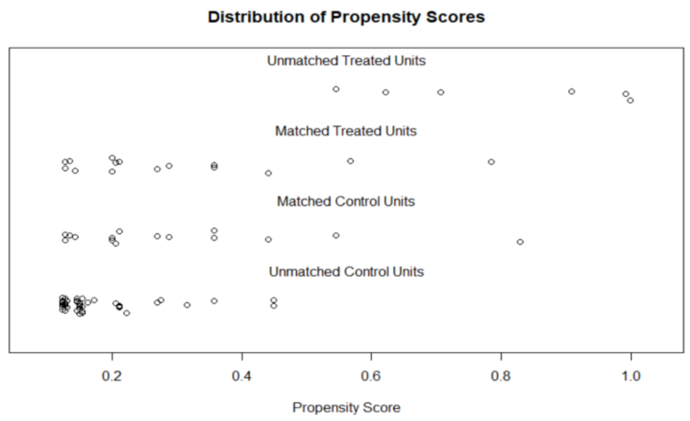

b)

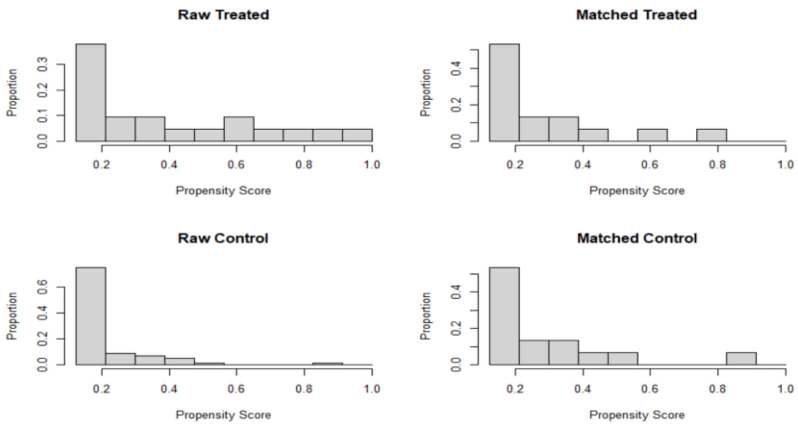

Supplemental figure 3: Quality assessment of propensity score matching

Distribution of propensity scores (a) in matched and unmatched patients with Ishak fibrosis ≥ F2 (“Treated Units”) and without fibrosis (“Untreated units”). Proportion of patients with and without Ishak fibrosis stage ≥ F2 (“Treated” and “control” respectively) before (raw; overall cohort) and after matching (matched).

Supplemental Figure 4

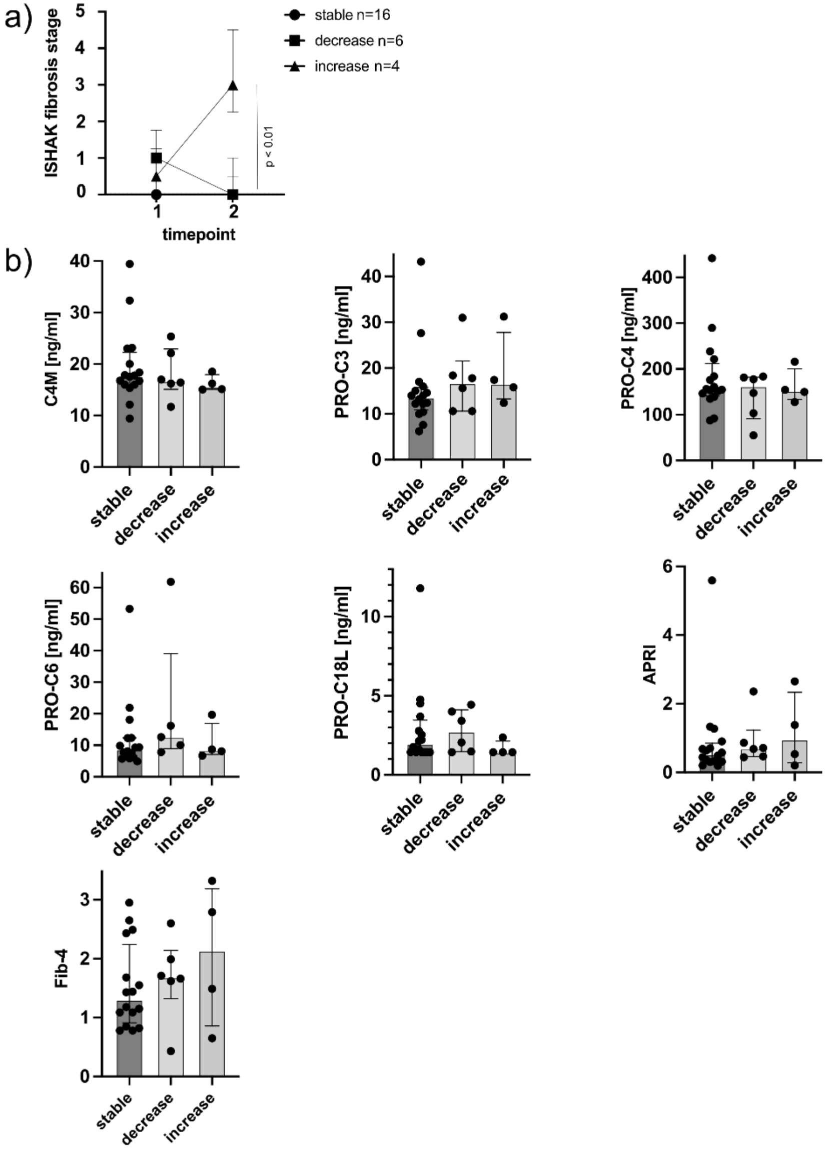

Supplemental figure 4: Assessment of predictive capacity of ECM biomarker levels for the development of fibrosis from year one samples

(a) Ishak fibrosis stage in patients with stable (dots), decreasing (square) or increasing (triangle) fibrosis stage between year 1 (=timepoint 1) and year 3-5 (=timepoint 2). Median and interquartile range (IQR) are shown. No significant differences were observed at timepoint 1. Patients with increasing fibrosis had significantly higher ISHAK fibrosis stage at timepoint two than patients with stable or decreasing fibrosis stage (Kruskal-Wallis test and Bonferroni adjustment for multiple comparisons). (b) Median levels of C4M, PRO-C3, PRO-C4, PRO-C6, PRO-C18L and median APRI and FIB-4 in patients with stable, decreasing or increasing ISHAK fibrosis stage between year 1 and year 3-5 are shown as bar charts with IQR. Differences between groups were assessed using Kurskal-Wallis test and Bonferroni post-hoc test and were not significant.

Supplemental Figure 5

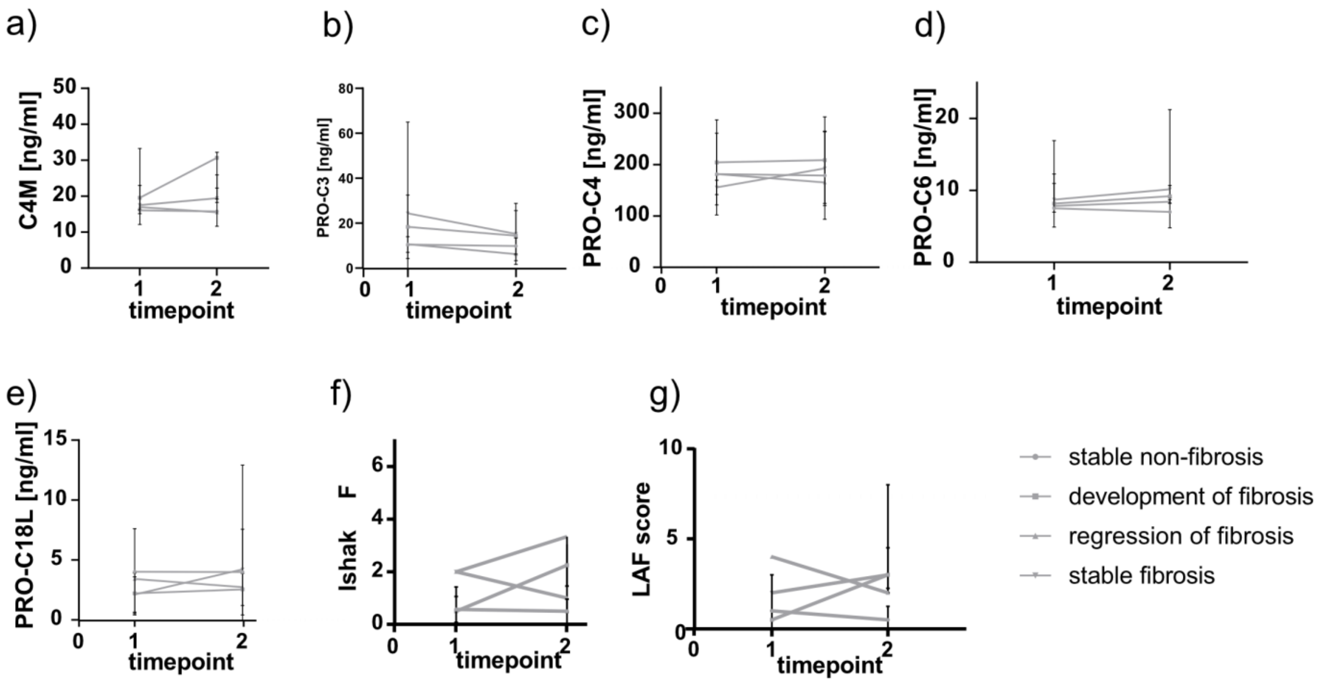

Supplemental figure 5: Longitudinal dynamics of ECM biomarker levels and fibrosis stages in serial patient samples

Dynamics of ECM biomarker levels (a-e), Ishak fibrosis stage (f) and LAF score (g) between first sampling (timepoint 1) and follow-up sampling (time-point 2). Median and IQR are shown. Stable non-fibrosis (n=7), development of fibrosis (n=4), regression of fibrosis (n=1), stable fibrosis (n=3).

Supplemental tables

Supplemental table1

De Long's test	**C4M**	**PRO-C3**	**PRO-C4**	**PRO-C6**	**PRO-C18L**	**APRI**	**FIB-4**	**C4M + PRO-C3**
**C4M**								
**PRO-C3**	0.95							
**PRO-C4**	0.70	0.75						
**PRO-C6**	0.59	0.63	0.88					
**PRO-C18L**	0.36	0.39	0.57	0.68				
**APRI**	**0.03**	**0.03**	0.06	0.09	0.21			
**FIB-4**	0.11	0.12	0.20	0.25	0.47	0.62		
**C4M + PRO-C3**	0.75	0.70	0.50	0.37	0.14	**0.003**	**0.03**	

Supplemental table 1: Comparison of AUCs between ECM biomarkers and non-invasive fibrosis scores

P-values of DeLong's test for the comparison of AUCs of different ECM biomarkers, APRI and FIB-4. Red background color indicates inferiority of the marker on the left side of the row in comparison to the marker at the top of the column. Green background color indicates superiority of the marker on the left side of the row in comparison to the marker at the top of the column. P-values <0.05 are regarded as significant and depicted in bold.

Supplemental table 2

	McNemar	specificity									
		**C4M**	**PRO-C3**	**PRO-C4**	**PRO-C6**	**PRO-C18L**	**APRI**	**APRI CutOff 1**	**FIB-4**	**FIB-4 CutOff 1.45**	**FIB-4 CutOff 3.25**
**sensitivity**	**C4M**		0.68	**0.02**	**0.001**	1.00	0.54	0.12	0.33	0.07	**< 0.001**
	**PRO-C3**	0.75		0.18	**0.001**	0.71	0.12	**0.01**	0.06	0.09	**< 0.001**
	**PRO-C4**	**0.03**	0.29		0.50	0.07	**0.01**	**0.001**	**0.004**	1.00	**< 0.001**
	**PRO-C6**	n/a	n/a	n/a		**0.001**	**< 0.001**	**< 0.001**	**< 0.001**	0.36	**< 0.001**
	**PRO-C18L**	0.45	1.00	0.45	n/a		0.60	0.25	0.38	0.09	**< 0.001**
	**APRI**	0.24	0.06	**0.004**	n/a	**0.02**		0.13	0.75	**< 0.001**	**0.001**
	**APRI CutOff 1**	**0.02**	**0.002**	**< 0.001**	n/a	**0.001**	0.06		0.75	**< 0.001**	**0.006**
	**FIB-4**	0.26	0.08	**0.008**	n/a	**0.049**	1.00	0.18		**< 0.001**	**< 0.001**
	**FIB-4 CutOff 1.45**	0.48	0.21	**0.02**	n/a	0.12	0.69	**0.04**	0.50		**< 0.001**
	**FIB-4 CutOff 3.25**	**0.01**	**0.001**	**< 0.001**	n/a	**0.001**	0.07	1.00	**0.03**	**0.008**	

Supplemental table 2: Comparison of sensitivies and specificities between ECM biomarker levels and non-invasive fibrosis scores

P-values of McNemar test for the comparison of sensitivities (below the black diagonal) and specificities (above the black diagonal) of different ECM biomarkers, APRI and FIB-4. Red background color indicates inferiority of the marker on the left side of the row in comparison to the marker at the top of the column. Green background color indicates superiority of the marker on the left side of the row in comparison to the marker at the top of the column. P-values <0.05 are regarded as significant and depicted in bold. E.g., PRO-C4 is significantly more sensitive than C4M while C4M is more specific than PRO-C4.

**Supplemental table 3**	All	F<2	F≥2	p-value
patient number	77	56	21	
age [years] (median (range))	52 (20 - 69)	51 (20 - 69)	57 (22 - 67)	0.12
female sex [%]	24 (31.2)	16 (28.6)	8 (38.1)	0.42
time after liver transplantation [months] (median (range))	23 (5 - 298)	12.5 (5 - 116)	48 (6 - 298)	**0.001**
AST [times upper limit of normal] (median (range))	0.8 (0.3 - 1.5) (n=76)	0.7 (0.3 - 1.5) (n=55)	0.9 (0.5 - 1.5)	**0.048**
ALT [times upper limit of normal] (median (range))	0.5 (0.1 - 1.8)	0.5 (0.1 - 1.5)	0.4 (0.2 - 1.8)	0.73
ALP [times upper limit of normal] (median (range))	0.8 (0.2 - 1.9) (n=73)	0.7 (0.2 - 1.8) (n=52)	1.1 (0.6 - 1.9)	**< 0.001**
gGT [times upper limit of normal] (median (range))	0.6 (0.2 - 13.0)	0.5 (0.2 - 13.0)	1.4 (0.3 - 11.8)	**0.008**
bilirubin [times upper limit of normal] (median (range))	0.5 (0.2 - 2.5)	0.5 (0.2 - 2.5)	0.6 (0.2 - 1.3)	0.52
platelets [/nl] (median (range))	178 (43 - 394)	178 (70 - 394)	184 (42 - 324)	0.80
creatinine [µmol/l] (median (range))	96 (58 - 242) (n=75)	99 (63 - 206) (n=74)	94 (58 - 242)	0.51
APRI Score (median (range))	0.4 (0.2 - 2.6) (n=76)	0.4 (0.2 - 1.4) (n=55)	0.5 (0.2 - 2.6)	0.27
FIB-4 Score (median (range))	1.4 (0.4 - 6.5) (n=76)	1.3 (0.4 - 3.3) (n=55)	1.4 (0.7 - 6.5)	0.15
Histopathological characteristics				
TCMR [n (%)]	0 (0.0)	0 (0.0)	0 (0.0)	n/a
subTCMR [n (%)]	39 (50.6)	25 (44.6)	14 (66.7)	0.13
indeterminate [n (%)]	19 (24.7)	12 (21.4)	7 (33.3)	0.37
NHR [n (%)]	19 (24.7)	19 (33.9)	0 (0.0)	**0.001**
RAI (median (range))	3 (0 - 8)	2 (0 - 6)	4 (1 - 8)	**0.005**
mHAI (median (range))	2 (0 - 7)	2 (0 - 6)	4 (2 - 7)	**< 0.001**
DSA positive/n (%)	32 (41.6)	19 (33.9)	13 (61.9)	**0.04**
Reason for LT				
AILD [n (%)]	25 (32.5)	19 (33.9)	6 (28.6)	0.79
alcoholic [n (%)]	15 (19.5)	9 (16.1)	6 (28.6)	0.33
NASH [n (%)]	1 (1.3)	1 (1.8)	0 (0.0)	1.00
HCC [n (%)]	2 (2.6)	2 (3.6)	0 (0.0)	1.00

viral [n (%)]	9 (11.7)	7 (12.5)	2 (9.5)	1.00
cryptogenic [n (%)]	13 (16.9)	9 (16.1)	4 (19.0)	0.74
other [n (%)]	12 (15.6)	9 (16.1)	3 (14.3)	1.00
TAC [n (%)]	32 (41.6)	28 (50.0)	4 (19.0)	**0.02**
CSA [n (%)]	41 (53.2)	26 (46.4)	15 (71.4)	0.07
EVR [n (%)]	1 (1.3)	1 (1.8)	0 (0.0)	1.00
SIR [n (%)]	2 (2.6)	1 (1.8)	1 (4.8)	0.47
C4M [ng/ml] (median (range))	17.8 (9.4 - 39.4)	16.9 (9.4 - 37.7)	20.1 (15.1 - 39.4)	**0.002**
PRO-C3[ng/ml] (median (range))	11.8 (4.0 - 42.2)	11 (4 - 42.2)	15.8 (7.4 - 24.6)	**< 0.001**
PRO-C4 [ng/ml] (median (range))	161.5 (54.8 - 442.0) (n=76)	152.0 (54.8 - 411.5)	179.7 (140.0 - 442.0) (n=20)	**0.004**
PRO-C6 [ng/ml] (median (range))	8.5 (4.0 - 21.9) (n=75)	7.7 (4.0 - 21.9) (n=55)	10.0 (7.8 - 21.2) (n=20)	**0.003**
PRO-C18L [ng/ml] (median (range))	2.2 (1.4 - 47.7) (n=75)	2.0 (1.4 - 47.7) (n=54)	2.9 (1.4 - 15.9)	0.06

Supplemental table 3: Demographics from patients with surveillance liver biopsies

Patients' demographics for the cohort with ALP, AST and ALT below 2 times upper limit of normal, stratified according to presence of fibrosis ≥F2 (ISHAK fibrosis staging). Significant p-values for comparison between fibrosis and non-fibrosis group are designated in bold. Mann-Whitney-U Test was used for comparison of continuous variables and Fisher's exact test was used for the comparison of categorial variables between these two groups. P-values <0.05 were considered significant.

**Supplemental table 4**	AUC	SE	p-value	95% CI	Cut-Off	Sensitivity (95% CI) [%]	Specificity (95% CI) [%]	Accuracy (95% CI) [%]	PPV (95% CI) [%]	NPV (95% CI)	LR+ (95% CI)	LR- (95% CI)
C4M	0.67	0.07	**0.04**	0.53 - 0.82	19.0	61.9 (38.4 - 81.9)	**76.8 (63.6 - 87.0)**	**73.7 (62.5 - 83.1)**	40.9 (27.9 - 55.3)	**88.6 (81.6 - 93.2)**	2.67 (1.49 - 4.78)	0.50 (0.28 - 0.87)
PRO-C3	0.78	0.07	**0.001**	0.65 - 0.91	13.8	66.7 (43.0 - 85.4)	80.4 (67.6 - 89.8)	**77.5 (66.6 - 86.3)**	46.8 (32.4 - 61.8)	**90.3 (83.4 - 94.5)**	**3.39 (1.84 - 6.25)**	0.41 (0.22 - 0.77)
PRO-C4	0.69	0.07	**0.02**	0.56 - 0.82	155.3	**80.0 (56.3 - 94.3)**	57.1 (43.2 - 70.3)	61.9 (50.0 - 72.8)	32.6 (25.0 - 41.3)	**91.7 (81.7 - 96.5)**	1.87 (1.28 - 2.71)	0.35 (0.14 - 0.87)
PRO-C6	0.76	0.06	**0.002**	0.64 - 0.88	7.7	**100.0 (83.2 - 100.0)**	50.9 (37.1 - 64.7)	61.0 (49.1 - 72.1)	34.6 (28.8 - 40.9)	**100.0**	2.04 (1.56 - 2.67)	**0.00**
PRO-C18L	0.62	0.08	0.17	0.45 - 0.78	2.3	71.4 (47.8 - 88.7)	**70.4 (56.4 - 82.0)**	**70.6 (59.0 - 80.6)**	38.5 (27.7 - 50.6)	**90.5 (82.5 - 95.0)**	2.41 (1.47 - 3.94)	0.41 (0.20 - 0.82)
APRI	0.59	0.09	0.28	0.41 - 0.77	0.7	38.1 (18.1 - 61.6)	**89.1 (77.8 - 95.9)**	**78.6 (67.7 - 87.2)**	47.5 (26.3 - 69.7)	**84.7 (79.7 - 88.7)**	**3.49 (1.38 - 8.86)**	0.69 (0.49 - 0.98)
APRI CutOff 1					1.0	14.3 (3.1 - 36.3)	**96.4 (87.5 - 99.6)**	**79.5 (68.6 - 87.9)**	50.5 (15.5 - 85.0)	**81.3 (78.3 - 83.9)**	**3.93 (0.71 - 21.88)**	0.89 (0.74 - 1.07)
FIB-4	0.62	0.09	0.16	0.44 - 0.79	2.0	42.9 (21.8 - 66.0)	**85.5 (73.3 - 93.5)**	**76.7 (65.6 - 85.6)**	43.3 (25.4 - 63.2)	**85.2 (79.7 - 89.5)**	2.95 (1.31 - 6.62)	0.67 (0.45 - 0.98)
FIB-4 CutOff 1.45					1.45	47.6 (25.7 - 70.2)	60.0 (45.9 - 73.0)	57.5 (45.6 - 68.7)	23.6 (15.1 - 34.9)	**81.5 (73.6 - 87.5)**	1.19 (0.68 - 2.07)	0.87 (0.55 - 1.38)
FIB-4 CutOff 3.25					3.25	23.8 (8.2 - 47.2)	**96.4 (87.5 - 99.6)**	**81.4 (70.9 - 89.4)**	63.0 (26.3 - 89.0)	**83.0 (79.2 - 86.2)**	**6.55 (1.37 - 31.19)**	0.79 (0.62 - 1.01)

specificity

	McNemar	**C4M**	**PRO-C3**	**PRO-C4**	**PRO-C6**	**PRO-C18L**	**APRI**	**APRI CutOff 1**	**FIB-4**	**FIB-4 CutOff 1.45**	**FIB-4 CutOff 3.25**
	**C4M**		0.82	**0.02**	**0.004**	0.61	0.17	**0.007**	0.36	0.15	**0.007**
	**PRO-C3**	1.00		**0.02**	**< 0.001**	0.38	0.23	**0.02**	0.58	**0.02**	**0.02**
	**PRO-C4**	0.13	0.45		0.71	0.23	**0.001**	**< 0.001**	**0.004**	0.85	**< 0.001**
**sensitivity**	**PRO-C6**	**n/a**	**n/a**	**n/a**		**0.004**	**< 0.001**	**< 0.001**	**< 0.001**	0.45	**< 0.001**
	**PRO-C18L**	0.69	1.00	0.69	**n/a**		0.08	**0.002**	0.19	0.29	**0.002**
	**APRI**	0.30	0.11	0.02	**n/a**	0.07		0.13	0.69	**< 0.001**	0.22
	**APRI CutOff 1**	**0.02**	**0.003**	**0.002**	**n/a**	**0.004**	0.06		**0.03**	**< 0.001**	1.00
	**FIB-4**	0.45	0.27	0.06	**n/a**	0.2	1.00	0.03		**< 0.001**	**0.03**
	**FIB-4 CutOff 1.45**	0.61	0.39	0.09	**n/a**	0.3	0.63	0.02	1.00		**< 0.001**
	**FIB-4 CutOff 3.25**	0.08	**0.02**	**0.008**	**n/a**	**0.0**	0.38	0.50	0.13	0.06	

Supplemental table 4: Test characteristics of ECM biomarkers and non-invasive fibrosis scores in patients with surveillance liver biopsies

Summary of test performance of ECM biomarkers, APRI and FIB-4 (upper table) and p-values of McNemar test (lower table)for the comparison of sensitivities (below the black diagonal) and specificities (above the black diagonal). Sensitivity, specificity, accuracy, PPV, NPV, LR+ and LR- are shown including the 95% confidence interval (CI). Values which CI are not including 0.5 are depicted in bold. P-values of AUC <0.05 were regarded as significant and are depicted in bold. SE: standard error; PPV: positive predictive value; NPV: negative predictive value; LR+: positive likelihood-ratio;

LR-: negative likelihood-ratio. In the lower table, red background color indicates inferiority of the marker on the left side of the row in comparison to the marker at the top of the column. Green background color indicates superiority of the marker on the left side of the row in comparison to the marker at the top of the column. P-values <0.05 are regarded as significant and depicted in bold. E.g., APRI using the cut-off of 1 is significantly more sensitive than C4M while C4M is more specific than APRI at that cut-off.

**Supplemental table 5**	All patients	Group F<2	Group F≥2	p-value
patient number	30	15	15	
age [years] (median (range))	53 (20 - 69)	50 (20 - 69)	58 (22 - 67)	0.13
female sex [%]	9 (30)	4 (26.7)	5 (33.3)	1.00
time after liver transplantation [months] (median (range))	25 (6 - 116)	25 (6 - 116)	25 (6 - 107)	1.00
AST (times upper limit of normal) (median (range))	0.8 (0.3 - 1.5)	0.8 (0.3 - 1.5)	0.8 (0.5 - 1.5)	1.00
ALT (times upper limit of normal) (median (range))	0.4 (0.2 - 1.8)	0.5 (0.2 - 1.1)	0.3 (0.2 - 1.8)	0.41
AP (times upper limit of normal) (median (range))	0.9 (0.2 - 1.8)	0.6 (0.2 - 1.3)	1.1 (0.6 - 1.8)	**0.01**
gGT [(times upper limit of normal) (median (range))	0.9 (0.2 - 11.8)	0.5 (0.2 - 4.0)	1.4 (0.3 - 11.8)	0.07
bilirubin (times upper limit of normal) (median (range))	0.6 (0.2 - 2.5)	0.5 (0.2 - 2.5)	0.6 (0.3 - 1.3)	0.51
platelets [/nl] (median (range))	173 (43 - 338)	170 (43 - 323)	179 (85 - 338)	0.78
creatinine [µmol/l] (median (range))	92 (58 - 792)	93 (58 - 792)	81 (64 - 135)	0.46

APRI Score (median (range))	0.5 (0.1 - 3.6)	0.5 (0.1 - 3.6)	0.4 (0.2 - 1.0)	1.00
FIB-4 Score (median (range))	1.6 (0.5 - 9.8)	1.4 (0.5 - 9.8)	1.7 (0.5 - 5.0)	0.33
Histopathological characteristics				
TCMR [n (%)]	0 (0)	0 (0)	0 (0)	n/a
subTCMR	18 (60)	7 (46.7)	11 (73.3)	0.26
indeterminate [n (%)]	7 (23.3)	3 (20.0)	4 (26.7)	1.00
NGF [n (%)]	5 (16.7)	5 (33.3)	0 (0)	**0.04**
RAI (median (range))	3 (0 - 8)	2 (0 - 6)	4 (1 - 8)	**0.03**
mHAI (median (range))	3 (0 - 7)	2 (0 - 6)	3 (2 - 7)	**0.01**
DSA positive [n (%)]	16 (53.3)	5 (33.3)	11 (73.3)	0.07
reason for LT				
AILD [n (%)]	11 (36.7)	5 (33.3)	6 (40.0)	1.00
alcoholic [n (%)]	7 (23.3)	2 (13.3)	5 (33.3)	0.39
viral [n (%)]	3 (10.0)	2 (13.3)	1 (6.7)	1.00
cryptogenic [n (%)]	4 (13.3)	2 (13.3)	2 (13.3)	1.00
other [n (%)]	5 (16.7)	4 (26.7)	1 (6.7)	0.33
TAC [n (%)]	9 (30)	7 (46.7)	2 (13.3)	0.11
CSA [n (%)]	21 (70)	8 (53.3)	13 (86.7)	0.11
EVR [n (%)]	0 (0)	0 (0)	0 (0)	n/a
SIR [n (%)]	0 (0)	0 (0)	0 (0)	n/a

Supplemental table 5: Demographics of patients matched for graft age

Patients' demographics for the cohort of patients that were propensity-score matched for time after transplantation. Significant p-values for comparison between fibrosis and non-fibrosis group are designated in bold. Mann-Whitney-U Test was used for comparison of continuous variables

and Fisher's exact test was used for the comparison of categorial variables between these two groups. P-values < 0.05 were considered significant.

**Supplemental table 6**	All	F<2	F≥2	p-value
patient number	26	18	8	
age [years] (median (range))	54 (21 - 64)	52 (21 - 63)	57 (22 - 64)	0.29
female sex [%]	9 (34.6)	7 (38.9)	2 (25.0)	0.67
time after liver transplantation [months] (median (range))	8 (2 - 12)	8 (2 - 12)	10 (2 - 12)	0.57
AST [times upper limit of normal] (median (range))	0.9 (0.5 - 7.2)	0.8 (0.5 - 7.2)	1.4 (0.5 - 4.4)	0.34
ALT [times upper limit of normal] (median (range))	1.0 (0.1 - 9.3)	0.8 (0.1 - 9.3)	1.5 (0.4 - 6.5)	0.37
ALP [times upper limit of normal] (median (range))	1.1 (0.2 - 8.0) (n=23)	1.0 (0.2 - 2.3) (n=16)	1.2 (0.7 - 8.0) (n=7)	0.38
gGT [times upper limit of normal] (median (range))	2.1 (0.2 - 38.7)	1.1 (0.2 - 11.8)	3.9 (0.3 - 38.7)	**0.047**
bilirubin [times upper limit of normal] (median (range))	0.5 (0.2 - 2.7)	0.5 (0.2 - 1.3)	0.6 (0.3 - 2.7)	0.46
platelets [/nl] (median (range))	174 (82 - 394)	180 (82 - 394)	167 (87 - 307)	0.77
creatinine [µmol/l] (median (range))	97 (61 - 792)	96 (63 - 792)	97 (61 - 137)	0.98
APRI Score (median (range))	0.6 (0.2 - 5.6)	0.6 (0.2 - 5.6)	0.7 (0.2 - 2.7)	0.37
FIB-4 Score (median (range))	1.5 (0.4 - 3.3)	1.5 (0.4 - 3.0)	1.6 (0.7 - 3.3)	0.72
Histopathological characteristics				
TCMR [n (%)]	5 (19.2)	4 (22.2)	1 (12.5)	1.00
subTCMR [n (%)]	10 (38.5)	8 (44.4)	2 (25.0)	0.42
indeterminate [n (%)]	7 (26.9)	2 (11.1)	5 (62.5)	**0.01**
NHR [n (%)]	4 (15.4)	4 (22.2)	0 (0.0)	0.28
RAI (median (range))	3 (0 - 8)	3 (0 - 8)	2.5 (1 - 6)	0.77
mHAI (median (range))	3 (0 - 6)	2 (0 - 6)	3.5 (1 - 6)	0.08

DSA positive/n (%)	11 (42.3)	6 (33.3)	5 (62.5)	0.22
reason for LT				
AILD [n (%)]	6 (23.1)	5 (27.8)	1 (12.5)	0.63
alcoholic [n (%)]	5 (19.2)	3 (16.7)	2 (25.0)	0.63
viral [n (%)]	5 (19.2)	4 (22.2)	1 (12.5)	1.00
cryptogenic [n (%)]	6 (23.1)	3 (16.7)	3 (37.5)	0.33
other [n (%)]	4 (15.4)	3 (16.7)	1 (12.5)	1.00
TAC [n (%)]	11 (42.3)	8 (44.4)	3 (37.5)	1.00
CSA [n (%)]	15 (57.7)	10 (55.6)	5 (62.5)	1.00
EVR [n (%)]	1 (3.8)	1 (5.6)	0 (0.0)	1.00
SIR [n (%)]	0 (0.0)	0 (0.0)	0 (0.0)	n/a
C4M [ng/ml] (median (range))	16.9 (9.4 - 39.4)	16.9 (9.4 - 32.3)	16.9 (15.1 - 39.4)	0.85
Pro-C3[ng/ml] (median (range))	14.3(6.2 - 43.2)	13.6 (6.2 - 31.0)	16.4 (10.6 - 43.2)	0.14
Pro-C4 [ng/ml] (median (range))	154.5 (54.8 - 442.0)	154.1 (54.8 - 289.7)	155.2 (127.6 - 442.0)	0.68
Pro-C6 [ng/ml] (median (range))	9.3 (4.9 - 61.9) (n=25)	9.3 (4.9 - 61.9) (n=17)	10.5 (6.7 - 19.7)	0.59
Pro-C18L [ng/ml] (median (range))	1.9 (1.44 - 11.8)	1.9 (1.4 - 11.8)	1.9 (1.4 - 4.8)	0.85

Supplemental table 6: Demographics of patients with sampling at one year after liver transplantation

Patients' demographics for the cohort of patients with samples taken one year after liver transplantation and available fibrosis outcome three to five years after liver transplantation, stratified according to presence of fibrosis ≥F2 (ISHAK fibrosis staging). Significant p-values for comparison

between fibrosis and non-fibrosis group are designated in bold. Mann-Whitney-U Test was used for comparison of continuous variables and Fisher's exact test was used for the comparison of categorial variables between these two groups. P-values < 0.05 were considered significant.
